# Distribution of inpatients with cardiovascular diseases and major depression

**DOI:** 10.1192/j.eurpsy.2021.911

**Published:** 2021-08-13

**Authors:** N. Kornetov, O. Molodykh, A. Arzhanik, N. Zvereva

**Affiliations:** 1 Department Of Psychiatry, Addiction And Psychotherapy, Siberian States Medical Unifersity, Tomsk, Russian Federation; 2 Department Of Psychiatry, Addiction And Psychotherapy, 5th year student of the medical faculty of the Siberian State Medical Unifersity, Seversk, Russian Federation; 3 Mathematics And Computer Science, Saint Petersburg State University, Tomsk, Russian Federation; 4 Department Of Cardiology, Sibirean State Scientific and Clinical Center of the Federal Medical and Biological Agency of Russia, Seversk, Russian Federation

**Keywords:** Depressive Disorder, Major Depression, Cardiovascular diseases, comorbidity

## Abstract

**Introduction:**

Major depression (MD) and anxiety symptoms (AS) are frequent cardiovascular diseases satellites (CVD).

**Objectives:**

To examine features of comorbid physical and mental disorders considering age and sex variability.

**Methods:**

Cross-sectional study 146 patients with СVD were examined in cardiologic department of the Medical Centre. Of these, 51 (60.0%) are women and 34 (40.0%) are men. Patients assessed the intensity of pain or its absence using Visual Analog Scale. Anhedonia was determined by the Snatch-Hamilton Pleasure Scale - SHAPS. A hospital scale, HADS, was used to assess anxiety and depression. The final clinical diagnosis of MD was carried out according to the DSM-V criteria. Quantitative and ordinal signs are presented in the form Me-Median (Q1; Q3) - the first and third quartiles, respectively.

**Results:**

The degree of MD among male and female p=0,17; in “A” and “B” groups р=0,4912. Among patients of “A” age group is 2 (Q1 1 ; Q3 4) p=0,1777 had no difference. Patients of group “B” scored 3 (Q1 1,0; Q3 5,0) р=0,0019. Anxiety among female is 9 (Q1 6,0; Q3 11,0), among male 7 (Q1 3,5; Q3 9,0) р=0,0006. In the group of patients under 60 years anxiety score is 8 (Q1 4,0; Q3 9,0), group above 60 - 8 (Q1 6,0; Q3 11,0) р=0,0045. Pain intensity scored 3 (Q1 1,0; Q3 5,0) among male, 5 (Q1 3,0; Q3 7,0) among female p=0,0009.

**Conclusions:**

Despite invariability of main depression symptoms among sex and partly age, pain and anxiety symptoms prevailed in elderly male and female.

